# Morphological and functional maturity of the oral jaws covary with offspring size in Trinidadian guppies

**DOI:** 10.1038/s41598-017-06414-6

**Published:** 2017-07-18

**Authors:** T. R. Dial, L. P. Hernandez, E. L. Brainerd

**Affiliations:** 1 Department of Ecology and Evolutionary Biology, Brown University, Providence, RI USA; 2 Department of Biological Sciences, The George Washington University, Washington, D.C. USA

## Abstract

Large size of individual offspring is routinely selected for in highly competitive environments, such as in low-predation populations of the Trinidadian guppy (*Poecilia reticulata*). Large guppy offspring outcompete their smaller conspecifics, but the functional mechanisms underlying this advantage are unknown. We measured jaw kinematics during benthic feeding and cranial musculoskeletal morphologies in neonates and juveniles from five populations of Trinidadian guppy and found that both kinematics and morphologies vary substantially with neonatal size. Rotation at the intramandibular joint (IMJ), but not the quadratomandibular joint (QMJ), increases with size among guppy offspring, from 11.7° in the smallest neonates to 22.9° in the largest neonates. Ossification of the cranial skeleton varies from 20% in the smallest neonates to 90% in the largest. Relative to standard length (SL; jaw tip to caudal fin base distance), the surface area of jaw-closing musculature scales with positive allometry (SL^2.72^) indicating that muscle growth outpaces body growth. Maximum gape also scales with positive allometry (SL^1.20^), indicating that larger neonates are capable of greater jaw excursions. These findings indicate that size is not the sole adaptive benefit to producing larger offspring; maturation provides a potential functional mechanism underlying the competitive advantage of large offspring size among Trinidadian guppies.

## Introduction

**Figure 1:**
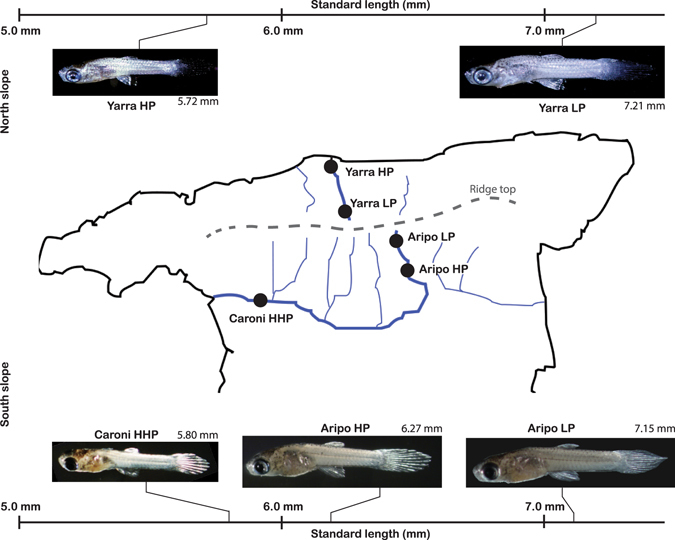
Geographic locations and average neonatal size among five Trinidadian guppy populations in the Northern Range Mountains. Repeated evolution of large offspring size has occurred in low-predation (LP) localities on both the northern (Yarra drainage) and the southern slopes (Caroni and Aripo drainages). The Caroni HHP exhibit an extremely high-predation (HHP) phenotype relative to Aripo HP. Standard length (SL) is defined as a straight line drawn from the tip of the lower jaw (mouth closed) to the base of the tail (caudal peduncle). Adobe Illustrator CS6 (Version 16.0.4; http://www.adobe.com/products/illustrator.html) was used to generate the map.

Competition tends to select for large size of individual offspring, balanced by a relatively smaller total number of offspring^1–8^. The competitive advantage of large offspring size is generally thought to occur during dominance interactions, such as during interference competition, in which organisms defend a resource and thus directly interfere with conspecifics^2, 9^. Interference competition can create a non-linear disparity between size and resource acquisition^2^, which has been shown to favor the evolution of larger offspring, with more developed weapons^10^. In contrast, exploitative competition^11, 12^, in which organisms indirectly compete over limited available resources, is not predicted to result in the evolution of size disparity among offspring; an organism’s effectiveness at obtaining limited resources should be proportional to its size alone^2, 13^. Despite this, the Trinidadian guppy (*Poecilia reticulata*) has shown repeated convergence of large offspring size in environments where individuals indirectly compete over limited trophic resources (Fig. 1)^3, 14, 15^.

The guppy is a species of live-bearing fish (Poeciliidae) that inhabits freshwater streams flowing from the Northern Range Mountains of Trinidad (Fig. 1). Guppies have repeatedly invaded and subsequently evolved within environments above waterfall barriers, which preclude large piscivorous fish from establishing^14, 16^. These low-predation (LP) environments are characterized by high guppy biomass and low invertebrate abundance, which force LP guppies to feed on encrusting algae and diatoms from the benthos^17^. Primary production within LP environments has been shown to constrain guppy population size, indicating that individuals indirectly compete over a limited supply of available food^18^. In such environments, large guppy offspring have higher fitness than their smaller high-predation (HP) counterparts^19^. It is likely that increased ability to exploit these limited resources, by more effectively or efficiently removing the encrusting food source, leads to an increased fitness advantage among larger offspring at birth.

**Figure 2:**
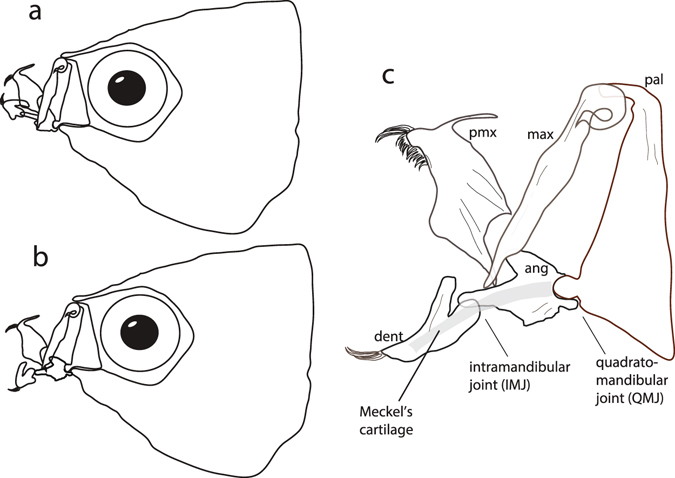
Schematic of the jaw while closed (**a**) and while applied to the substrate to scrape food (**b**). Close-up of the jaws (**c**) illustrates that upon full mouth opening much of the gape is due to rotation about the intramandibular joint (IMJ). The IMJ sits at the intersection of the angular and dentary bones, which are connected via Meckel’s cartilage (gray band running deep to the lower jaw). The IMJ is known to be mobile, with up to 90° of rotation, among adult poeciliids^21^. Abbreviations: pmx, premaxilla; max, maxilla; pal, palantine; dent, dentary; ang, angular.

Poeciliids as a group are specialized scrapers, possessing jaws adapted for removal of encrusting food items from the benthos^20–22^. The lower jaw of most ray-finned fishes (Actinopterygii), is comprised of two bones (angular and dentary) that have been shown to allow some torsion along the long axis of the mandible during suction feeding^23^. In poeciliids, however, this joint, termed the intramandibular joint (IMJ), allows for rotation about the medio-lateral axis of the lower jaw and adult poeciliids produce up to 90° of IMJ rotation (Fig. 2)^21^. The fitness advantages of mobility at the IMJ have been demonstrated through increased benthic scraping performance across several unrelated groups of fishes that all have highly mobile IMJs and feed by scraping^24, 25^. The IMJ allows for greater gape, enhanced bite force and the ability to close the mouth even while the oral jaws are protruded^20, 21, 24–27^. Among poeciliids, mobility at the IMJ correlates with reliance on encrusting food sources^21, 22^. It is not fully understood, however, the degree to which the joint functions in early life stages, particularly within the ecological context of newborn guppies competing over limited benthic resources.

The effect of offspring size on fitness (growth rate, time to first reproduction, etc.) in the guppy depends on the availability of resources. When resources are unlimited, smaller HP offspring experience catch up growth, where higher growth rates increase HP size to meet LP juvenile size by one month post natal^28, 29^. When guppy densities are high and resources are limiting, however, larger juveniles experience higher growth rates than their smaller counterparts, maintaining their size advantage throughout ontogeny^19^. The finding that larger offspring grow faster than smaller offspring when resources are limiting suggests a non-linear disparity between size and resource acquisition. It is possible that this disparity is the product of morphological features that confer a competitive advantage to the LP offspring. Since increased mobility at the IMJ among poeciliids correlates with improved performance when scraping encrusting food, we predict that LP offspring are born with greater mobility at the IMJ.

From high-speed video of offspring feeding from the benthos, we will measure jaw joint kinematics among five populations of Trinidadian guppies. We predict that LP offspring will exhibit increased function of their feeding apparatus, as evidenced by increases in intramandibular joint rotation, quadratomandibular joint rotation and total gape. By applying immunohistochemical and traditional histological staining techniques to visualize the musculoskeletal system, we will quantify morphological development among neonatal guppy head skeletons. To distinguish between size and population effects, we will collect data at two time points for each population: first at birth, where size is predicted to vary among populations; and second at later stages, where juvenile size ranges overlap. This functional and morphological investigation of neonates will offer insights into the advantage of large offspring size and will provide a better understanding of how size and maturity influence organismal growth and development, and how evolution shapes these parameters at the population level.

## Results

**Figure 3:**
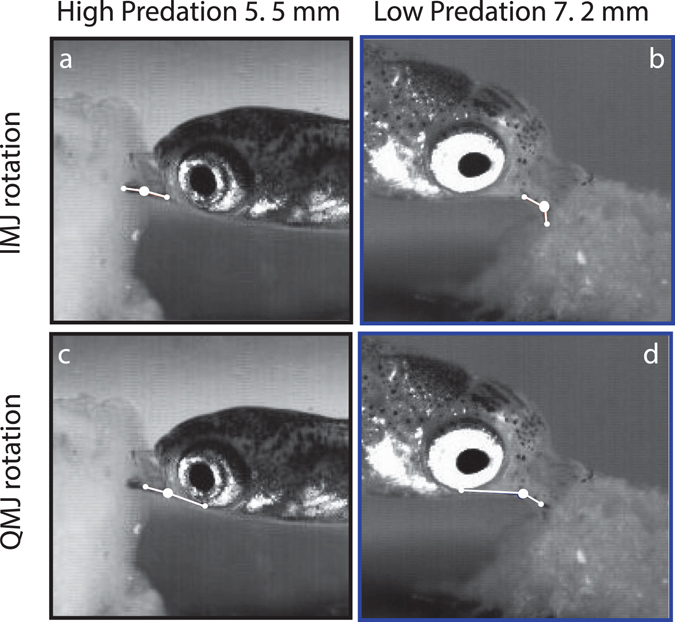
Feeding kinematics in neonates from HP and LP populations. Standard length of the two individuals is shown at top. (**a**,**b**) Intramandibular (IMJ) rotation angle between high-predation (**a**) and low-predation (**b**) neonates. (**c**,**d**) Quadratomandibular (QMJ) rotation between high (**c**) and low (**d**) predation neonates. Rotation at the IMJ, but not at the QMJ, increases with neonatal size.

A total of 45 neonatal offspring (n = 3 per brood, 3 broods per population, 5 populations) were filmed with high-speed video (Fig. 3) in their brood groups feeding on an encrusting food source (5–6 feeding events per brood). Following the feeding trials, neonates were euthanized and fixed for morphological analysis. Juveniles (n = 45) were reared for 10–20 days postnatal to obtain overlapping size ranges for all populations and the same filming and fixing procedures were followed. All kinematic data are reported as brood averages.

As expected from previous studies on guppy life histories^30–33^, standard length (Fig. 1) of guppy neonates was significantly different among populations (ANOVA: F (4, 40) = 394.73; p < 0.0001). Low-predation (LP) neonates were significantly larger than their high-predation (HP) counterparts across drainages (Tukey HSD: p < 0.0001).

### Feeding kinematics: neonates

**Figure 4:**
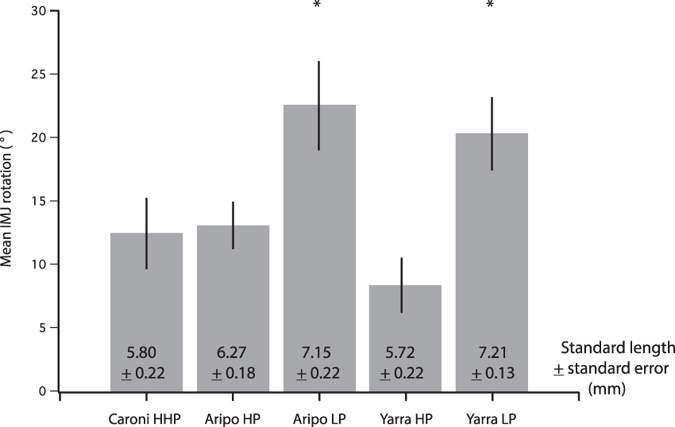
Mean intramandibular joint (IMJ) rotation among neonates for each population. Low-predation offspring exhibit nearly twice the IMJ mobility during feeding on encrusting food sources compared to their high-predation counterparts (asterisk indicates significance: p = 0.0007; error bars indicate standard error). Mean neonatal standard length (±standard error) indicated for each population. HHP, high-high-predation; HP, high-predation; LP, low-predation.

Guppy neonates showed substantial differences in IMJ rotation among populations (Fig. 4). There was a significant effect of population on intramandibular joint (IMJ) rotation (ANOVA: F (4, 10) = 12.23; p = 0.0007; Figs 3 and 4). Post-hoc Tukey-HSD indicates that the two LP populations have greater IMJ mobility than their HP counterparts. The neonates from the LP populations are also significantly longer at birth (Fig. 4). Quadratomandibular joint (QMJ) rotation showed no significant difference among population (ANOVA: F (4, 10) = 2.81; p = 0.085).

### Feeding kinematics: neonates and juveniles

**Figure 5:**
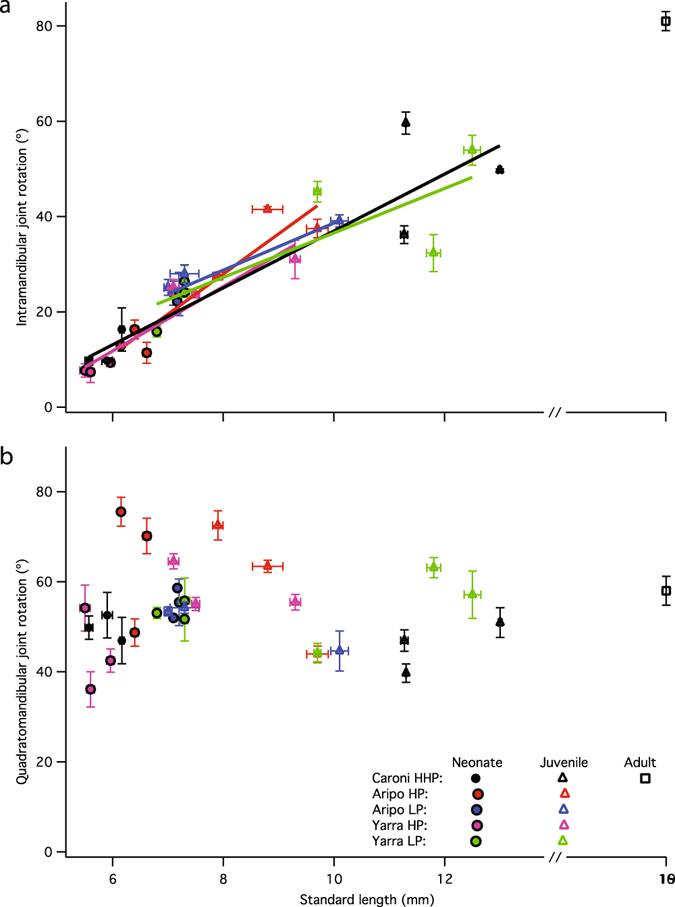
Intramandibular and quadratomandibular joint rotation in relation to body length in neonates and juveniles. Intramandibular joint rotation (**a**) increases with size among neonates and throughout ontogeny, but quadratomandibular joint rotation (**b**) does not change with size. Five populations and two age classes are presented (neonates indicated by closed symbols; juveniles indicated by open triangles; one adult indicated by open square for reference). Each data point shows mean brood joint rotation and mean standard length + standard error for 5–6 scraping events per brood. Regression lines in A are distinct from linear mixed model reported in Results.

Among neonates and juveniles examined herein, size explained a significant amount of variation in jaw kinematics (Fig. 5). Mobility at the IMJ is significantly and positively correlated with standard length (Linear regression: R^2^ = 0.82; F (1, 28) = 126.36; p < 0.0001). This trend continues to increase with size where 12 mm juveniles produce upwards of 50 degrees of rotation and adults (>20 mm) produce upwards of 90 degrees of rotation (Fig. 5a).

Regression analyses for individual populations indicate that within each population, IMJ rotation increases with increasing standard length (Fig. 5a): Aripo HP: R^2^ = 0.88; F (1, 5) = 30.52; p < 0.0052; Aripo LP: R^2^ = 0.92; F (1, 5) = 43.20; p < 0.0028; Caroni HHP: R^2^ = 0.85; F (1, 5) = 24.01; p < 0.0080; Yarra HP: R^2^ = 0.90; F (1, 5) = 35.04; p < 0.0041; Yarra LP: R^2^ = 0.67; F (1, 5) = 8.10; p < 0.0466.

To determine the effect of population on the data, an ANCOVA with standard length as the covariate was performed. Prior to conducting the ANCOVA, we found no interaction between our independent variable (population) and covariate (standard length; p = 0.5573). ANCOVA results indicate that population was not a significant predictor of IMJ mobility when taking size into account (p = 0.7189), therefore we modeled population as a random effect with size as a fixed effect and ran a linear mixed model to determine the significance of size in predicting IMJ rotation. Linear mixed effects model with population as the random effect indicated that standard length accounts for a significant amount of variation in IMJ rotation (p < 0.0001).

Quadratomandibular joint rotation does not change with size throughout the ontogenetic series examined herein (Linear regression: R^2^ = 0.01; F (1, 28) = 0.25; p = 0.62; Fig. 5b). Population does not explain variation in QJR across the size series examined herein (ANCOVA (F (5, 24) = 1.8828; p = 0.1349; interaction pop*SL p = 0.1868). Linear mixed effects model with population as the random effect and size as the fixed effect indicated that standard length does not account for a significant amount of variation in QMJ rotation (p = 0.707).

### Maximum gape

**Figure 6:**
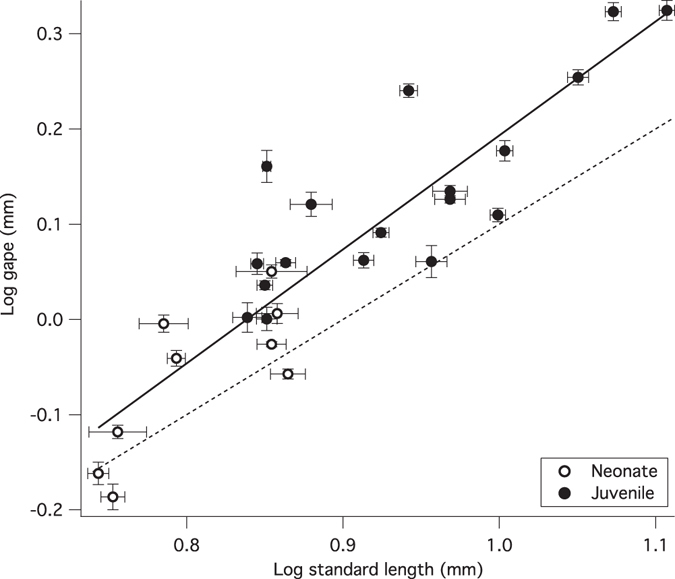
Positive allometric scaling of maximum gape among guppy offspring. Neonates are shown in open circles, postnatal juveniles shown in closed circles. If gape increased isometrically with body length, the points would fall along the dotted line with a slope of 1. Positive allometry indicates maximum gape is relatively and absolutely greater in larger fish. Each data point indicates mean brood gape (n = 5–6 scraping events per brood) and standard length + standard error.

Maximum gape while feeding increases with positive allometry among guppy neonates, scaling on a log-transformed plot with a slope of 1.20 ± 0.12 (R^2^ = 0.80; F (1, 52) = 101.5; p < 0.0001; Fig. 6). A scaling exponent significantly greater than 1 indicates that the linear distance between the oral jaws during maximum gape is relatively greater in larger offspring. The scaling of gape size among guppy offspring (SL^1.2^) indicates larger offspring not only take larger absolute bites, but they also take relatively larger bites.

### Head ossification

**Figure 7:**
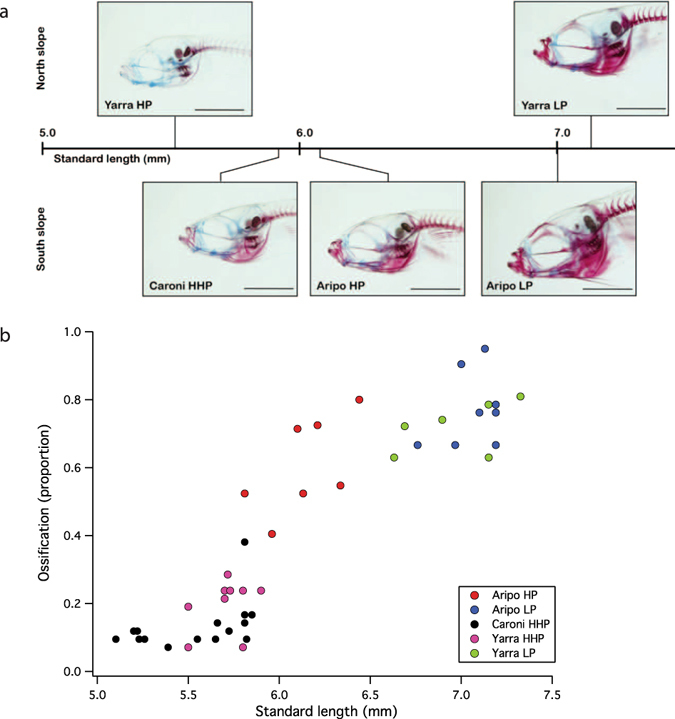
(**a**) Guppy neonates exhibit substantial variation in ossification of the head skeleton at birth. Cleared and stained neonatal specimens indicate cartilage (blue stain) and bone (red), from each of the five populations studied, aligned by standard length. North and South slope of the Northern Range Mountains in Trinidad indicated. Scale bar = 1 mm. All images to scale. (**b**) Development of the skull (as assessed by cranial ossification) increases rapidly with size among neonates. Of 18 skeletal elements examined within the head, the smallest neonates are born with fewer than 20% of these elements ossified. Neonates from LP environments are born ~20% larger, but with 400% greater ossification within the head skeleton. Populations are coded by color.

The degree of cranial ossification observed in neonatal guppies varied substantially among neonates (Fig. 7a). The smallest neonates (Caroni HHP and Yarra HP) are born with fewer than 20% of the bones in their head skeleton showing evidence of ossification (red staining), whereas the larger, low-predation neonates (Aripo LP and Yarra LP) are born with over 90% of their head skeleton ossified (Fig. 7b). The increase in neonatal head ossification is steep among populations, such that a 20% increase in size yields over four-fold increase in skeletal ossification (Fig. 7b). Ossification of the following skeletal elements distinguishes LP neonates from their HP counterparts: angular, hyomandibula, quadrate, ceratohyal and autopalatines. Caroni HHP and Yarra HP neonates lack ossification of these key elements, whereas Aripo LP and Yarra LP neonates possess full ossification of these elements. The largest juvenile guppies cleared and stained (10 mm) show that head ossification plateaus around 95% (the hypohyal and basihyal elements remain cartilaginous into adulthood).

### Adductor mandibulae scaling

**Figure 8:**
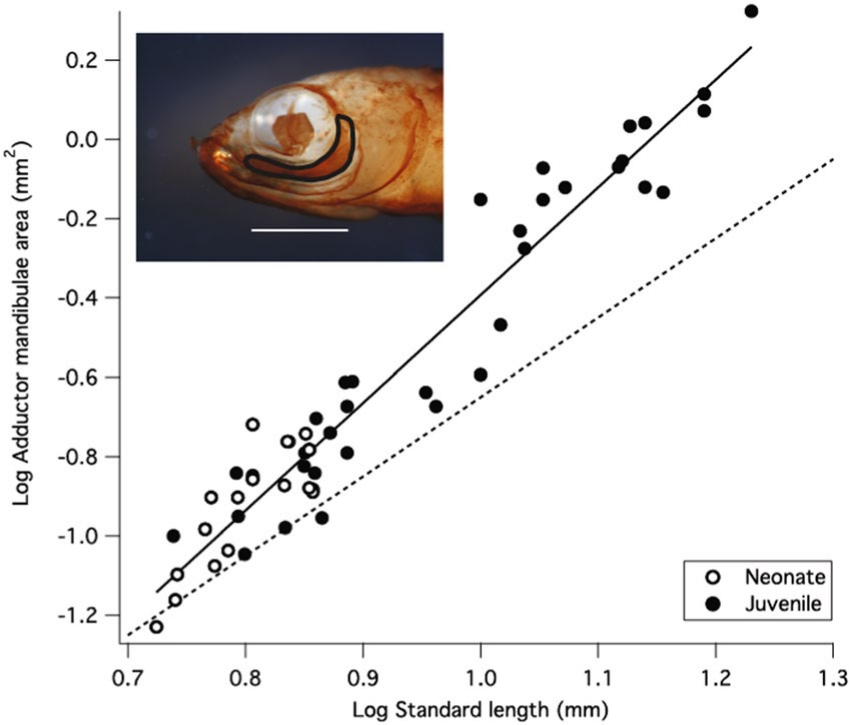
Positive allometric scaling of surface area of the adductor mandibulae relative to body length. Inset shows adductor mandibulae muscle area outlined in black (scale bar = 1 mm). If area of the adductor mandibulae increased isometrically, the points would fall along the dotted line with a slope of 2, which indicates isometry of surface area relative to standard length. Positive allometry indicates that growth of the adductor mandibulae outpaces growth of the body.

The surface area of the guppy adductor mandibulae scales with significant positive allometry (Fig. 8), such that larger guppy offspring possess relatively larger jaw-closing musculature. Adductor mandibulae area among neonatal individuals and juveniles increases with positive allometry throughout the first month of guppy postnatal development (up to 13 mm SL). The regression line of log adductor mandibulae area against log standard length yields a slope of 2.72 ± 0.10 (R^2^ = 0.94; F (1, 52) = 750.97; p < 0.0001), which is significantly higher than the scaling value expected for geometric similarity (isometry = 2).

## Discussion

We predicted that rotation at both the intramandibular and quadratomandibular joints would increase with neonatal size, but the data show that guppy offspring only exhibit a positive relationship between joint mobility and standard length at the intramandibular joint (IMJ; Fig. 5). Intramandibular joint rotation is positively allometric among neonates and postnatal juveniles, such that larger offspring employ greater movement at the IMJ while feeding (Figs 3, 4 and 5). This increase in joint rotation with size is correlated with a relative increase in maximal gape (Fig. 6). In other fishes, IMJ mobility has been shown to increase maximal gape as well as contact area of oral jaws with the substrate and force production^21, 24, 27^. In the guppy, the advantages associated with increased IMJ mobility might lead to the increase in scraping performance observed among larger offspring^19^.

An increase in mobility at the IMJ is unlikely due to changes of size per se. Joint rotation should not change with organismal size, because the degree of rotation about any given joint depends on the potential excursion afforded by the joint itself, and not the length of the elements involved^34, 35^. The observed increase in IMJ mobility with size among guppy offspring (Fig. 5) suggests that the morphology of the lower jaw is different among neonates. The observed increase in IMJ mobility occurs concurrent with increases in ossification pattern and muscular development, two separate measures of maturity that indicate guppies are born at varying stages of morphological maturity.

Throughout ontogeny the guppy skeleton is ossified in a regular pattern^36^, and we find that populations of Trinidadian guppies are born at varying points along this developmental trajectory, with the largest neonates possessing the greatest degree of cranial ossification (Fig. 7). Over the size range of guppy neonates investigated in this study (5.5 mm – 7.2 mm), we observe a four-fold increase in the number of cranial elements that are ossified at birth (Fig. 7b). The cranial skeleton is nearly fully ossified among the largest offspring, and only a few additional elements ossify during postnatal development. Growth of the muscles responsible for scraping behaviors provides secondary evidence that guppy neonates are born at different stages of morphological maturity. Adductor mandibulae area scales with significant positive allometry among guppy neonates and among postnatal juveniles (Fig. 8). The positive allometric scaling of adductor mandibulae size indicates that the growth of this muscle outpaces the growth of the entire organism, a pattern consistent with the development of early stage morphologies among larval fishes^37^. These profound differences in degree of ossification and muscular growth provide evidence that guppy neonates are born at varying levels of morphological maturity, and we hypothesize that maturation of the mouth opening mechanism explains the observed positive allometry of IMJ rotation among neonates.

The mouth opening mechanism (i.e., jaw depression) in fishes continues to develop even after first feeding has initiated^38^. Several jaw depression mechanisms are thought to actuate the lower jaw, and in all cases, mouth opening is the product of morphological maturation of jaw depression musculature^39, 40^. One of the last jaw opening muscles to develop and become functional is the geniohyoideus (=protractor hyoideus), which extends from the hyoid apparatus (ceratohyals) to the tip of the lower jaw (dentary symphysis)^38^. This muscle is the most likely candidate for causing IMJ rotation in the guppy^38^ and we propose that its function in causing IMJ rotation develops over the range of neonates investigated in this study. We hypothesize that the geniohyoideus muscle is not fully developed in the smallest HP neonates; indeed, the certatohyals are not ossified until later stages. We suggest that guppy offspring are born across a range of developmental stages where maturation of the jaw opening system varies widely and influences jaw function and competitive ability of neonates.

The positive effects of size on musculoskeletal development and feeding kinematics suggests that larger guppy neonates in low-predation (LP) environments are better equipped to compete within the resource limited environments into which they are born. Competition experiments have shown that when resources are limiting, large guppy juveniles exhibit higher growth rates than their smaller counterparts^19^. The current finding that larger offspring are both more mature and possess increased IMJ mobility provides a potential mechanism underlying this competitive advantage. The advantage of producing larger offspring in low resource, high competition environments, where these larger and more developed offspring can more efficiently obtain^19^ and utilize^41^ limited resources, might help to maintain selection for the LP phenotype.

In the environmental context of limited resources, the LP, high competition guppy phenotype has evolved repeatedly and rapidly^16^. Female guppies are selected to provision more yolk per ova, gestate for a greater period of time and ultimately produce larger offspring, among other life history traits^42^. Here, we provide evidence that associated with these life history features are morphological and functional traits important for exploitative competition. Low-predation guppy offspring are born not only larger, but also more mature and with more jaw mobility than their smaller counterparts. The observed disparity between size and resource acquisition among guppy offspring competing over limited resources is perhaps the product of the additive effects of maturation and size. We suggest that size per se may not be the only adaptive benefit of producing larger offspring in highly competitive environments; large size may instead be of secondary importance to the maturation of morphological features that allow for enhanced foraging ability at birth.

## Methods

### Collection and housing

Pregnant female guppies were collected from five populations throughout Trinidad’s Northern Range Mountains (Fig. 1). Three populations were collected from the Caroni drainage, the major southern confluence draining westward into the Atlantic: Aripo low-predation (LP); Aripo high-predation (HP); and Caroni extreme high-predation (HHP). Two additional populations were collected from the Yarra River on the north slope of the mountain range: Yarra LP and Yarra HP. The predator communities differ between northern and southern slopes; the former contain mainly gobiid predators, while the latter contain a dominant cichlid (*Crenicichla alta*)^16, 43^. The selective pressures have been shown to be similar between slopes: high predation selects for accelerated life histories, while high competition in LP sites selects for prolonged life histories (i.e., larger offspring, longer gestation times, longer inter-brood intervals, later age of first reproduction, less overall investment in reproduction)^3, 14, 16, 31^.

Pregnant female guppies were housed within a field lab in isolated 2-liter tanks, where they later gave birth. Upon parturition, each mother was promptly removed to mitigate potential cannibalism and loss of the litter. Litter sizes were separated into five or fewer individuals per tank to standardize feeding amounts and other density effects. Fish were fed twice daily on a diet of *Artemia* nauplii in the morning and algae flakes in the evening. Tanks were housed within an open-air laboratory facility and exposed to ambient light, which maintained light:dark schedule at approximately 12:12 hours.

All procedures were approved by the Brown University Institutional Animal Care and Use Committee (Protocol #: 1211035 to E. L. Brainerd). All experiments were performed in accordance with relevant guidelines and regulations.

### High-speed video

Filming of offspring while feeding was staged within a separate arena, equipped with lights, camera, and small chamber. The tank itself was constructed of acrylic (32 × 121 mm base) and filled with stock water to 50 mm height. Individuals were allowed a minimum of 30 minutes acclimation time prior to filming. All individuals were fasted the night prior to the feeding trial to facilitate feeding. Fish were fed a gelatin substance composed of dried brine shrimp, fruit, calcium and gelatin powder (hereafter referred to as ‘gel’). A small bolus of gel (approx. 0.1 g) was pressed onto a small, flat rock, and allowed to adhere for 5 minutes before being placed at the edge of the acrylic panel closest to the camera.

Video sequences were captured using a Photron FASTCAM 1024PCI (Photron USA, Inc., San Diego, CA, USA) fitted with a Nikon 105 mm, f/2.8 macro lens (Nikon Inc., Melville, NY, USA). Video was captured at 500 frames per second, at 1/1000 s shutter speed. The filming setup was illuminated with two LED lights (2.0 amp, 28 volt; Visual Instrumentation Corp., Lancaster, CA, USA).

Each filming event consisted of three individuals from each brood feeding concurrently upon the encrusting substrate (individual fish did not readily feed when isolated within the filming chamber). Filming sessions lasted on average between 30–60 minutes, which allowed 5–6 scraping events to be captured per brood. Data was collected from three broods per population, five populations and at two time points. This resulted in a total of n = 45 neonates, n = 45 juveniles and n = 5 adult females. Individuals were sacrificed at the end of each trial.

Following the feeding trials, guppies were sacrificed by overdose of tricaine methanesulfonate (Tricaine-S, Western Chemical Inc., Ferndale, WA, USA). Specimens were fixed in 4% buffered paraformaldehyde (Sigma, St. Louis, MO, USA) overnight and transferred to 70% EtOH for long-term storage.

### Staining

Skeletons of the guppy offspring were differentially stained and the bodies cleared to enable us to characterize and quantify degree of head skeletal ossification. The use of this technique stains cartilage blue and bone red. Bone and cartilage stains were performed by dehydrating specimens in 100% EtOH for 30 minutes prior to transfer into alcian blue solution (80% ethanol, 20% acetic acid) overnight. Specimens were rehydrated through a graded series of ethanol solutions (30 minutes in each) and were then placed overnight in a neutralizing solution of saturated sodium borate. They were bleached in a 30–35% H_2_O_2_ per 50 ml of 0.5% potassium hydroxide (KOH) for 1 hour, and then digested in a trypsin solution of 3 parts saturated sodium borate solution to 7 parts distilled H_2_O (10 g trypsin per liter) for 1 hour. Specimens were transferred to a solution of alizarin red and 0.5% KOH overnight, and finally cleared through a graded series (1:3, 1:1, 3:1) of glycerol:KOH before being imaged in 3:1 glycerol:KOH. Specimens were visualized using a Nikon dissecting microscope (Nikon SMZ800 dissecting scope and Nikon DXM1200C digital camera).

To determine the degree of muscle development during ontogeny, neonates were immunostained with the muscle-specific antibody MF-20, visualized after performing HRP color reaction and the perimeter of the adductor mandibulae traced from a lateral view. Specimens were skinned to promote penetration of the antibodies, were washed in phosphate buffered saline (PBS) + tween (PBST) and then bleached overnight in 2:1 Dent’s fixative:H_2_O_2_ (Dent’s fixative = 4 parts methanol to 1 part dimethyl sulfoxide). Specimens were washed in PBST and then treated with Proteinase K (10 μl/ml PBS) for 20 min, washed again in PBST and blocked in PBST/bovine albumin/goat serum (PBN) for two hours before being incubated in MF-20 primary antibody (DSHB, Iowa, USA) overnight at 20 °C. Specimens were washed a third time in PBST and blocked in PBN (2 hours) prior to application of horseradish peroxidase (HRP) secondary antibody (1:200 dilution; Promega Corp., Madison, WI, USA) overnight. Color reaction was performed using 3,3′-Diaminobenzidine (DAB chromogen kit, Biocare Medical, Concord, CA, USA) and specimens were imaged in 1:1 glycerol:PBS solution under a dissecting microscope (Nikon SMZ800 dissecting scope and Nikon DXM1200C digital camera).

### Analysis

Quantification of ossification within the cranial skeleton was performed by identifying (and assigning a value) to the presence (1), partial presence (0.5) or absence (0) of alizarin red uptake within each of the following feeding-associated skeletal elements: pharyngeal jaws, premaxillae, acrodont teeth, dentaries, hyomandibula, opercles, angulars, maxillae, prevomer, quadrate, urohyal, epihyal, ceratohyal, frontal, prefrontal-lateral ethmoids, autopalatines, hypohyal, basihyal. Ossification of cranial elements is presented as % of total head skeleton ossified, which was quantified by determining the mean of the values for presence (1), partial presence (0.5) or absence (0) of red coloration in all 18 elements measured in the head skeleton. This type of averaging yields, for example, a skeleton with 9 elements fully ossified (9 × 1 = 9) and 9 elements partially ossified (9 × 0.5 = 4.5) a % ossification of 75% (13.5/18 * 100).

The surface area of the superficial adductor mandibulae muscles was measured using IMAGEJ v.1.42 (National Institutes of Health, Bethesda, MD, USA). The stained muscle was outlined for each specimen to determine area. These values were log-transformed and regressed against log standard length to yield scaling relationships.

Kinematic variables during scraping of the gel substrate were quantified using IMAGEJ v.1.42 (National Institutes of Health, Bethesda, MD, USA). Linear gape distance was defined as a straight line connecting the rostral-most tip of the premaxillary and dentary bones. Quadratomandibular joint (QMJ) rotation was defined as a line extending rostrally from the ventral margin of the orbit to the QMJ, a line drawn down the length of the angular-articular bone complex and a vertex where these two lines intersect. Intramandibular joint (IMJ) rotation was defined as a line extending rostrally from the QMJ along the angular-articular bone complex, a line extending rostrally from the IMJ along the dentary to the point of lower jaw contact with the substrate and a vertex where these two lines intersect (Fig. 3). Quadratomandibular joint rotation was referenced to the closed position of the jaw (defined as a line extending rostrally from the ventral-most position of the orbit to the QMJ, a line drawn along the ventral margin of the lower jaw from the QMJ rostrally to the tip of the dentary during closed mouth and a vertex where these two lines intersect; Fig. 3). IMJ rotation was referenced to the closed jaw position of the lower jaw (i.e., a straight line, equaling 180°). Kinematic analysis was performed on several feeding trials per filming event and joint angles were each measured five separate times and averaged to yield the reported joint angle (standard error of five measures did not exceed ± 1 degree).

### Statistical analysis

To determine scaling relationships morphological variables were log-transformed and regression lines (reduced major axis) fit against log standard length. The resultant scaling coefficients were compared against expected isometric scaling values for each variable. Kinematic data were presented as brood averages. Regression analyses were performed on kinematic variables, fitted against standard length. One-way analyses of variance (ANOVA) were performed on kinematic variables with population as the factor. Where relevant, Tukey HSD post hoc tests were performed to identify means that are significantly different from each other. Analysis of covariance (ANCOVA) analyses were used to control for the effect of size among populations (an interaction analysis was first performed to determine homogeneity of slopes). Linear mixed effects models were performed to account for random effects of population. All statistical analyses were performed using JMP v.11 (SAS Institute, Cary, NC, USA).

### Data accessibility

Cranial morphology and kinematic data among populations of neonatal guppies will be deposited in the Dryad Digital Repository. Videos will be available on the Zoological Motion Analysis (ZMA) Portal.
